# A rapid co-culture stamping device for studying intercellular communication

**DOI:** 10.1038/srep35618

**Published:** 2016-10-18

**Authors:** Amin Hassanzadeh-Barforoushi, Jonathan Shemesh, Nona Farbehi, Mohsen Asadnia, Guan Heng Yeoh, Richard P. Harvey, Robert E. Nordon, Majid Ebrahimi Warkiani

**Affiliations:** 1 School of Mechanical and Manufacturing Engineering, University of New South Wales, Sydney, NSW 2052, Australia; 2 Graduate School of Biomedical Engineering, University of New South Wales, Sydney, NSW 2052, Australia; 3 Department of Engineering, Faculty of Science, Macquarie University, Sydney, NSW 2109, Australia; 4 Developmental and Stem Cell Biology Division, Victor Chang Cardiac Research Institute, Sydney, NSW, 2010; St. Vincent’s Clinical School and School of Biotechnology and Biomolecular Science, University of New South Wales, Sydney, NSW 2052, Australia; 5 Australian Centre for Nanomedicine, University of New South Wales, Sydney, NSW 2052, Australia; Garvan Institute of Medical Research, Darlinghurst, Sydney, NSW 2010, Australia; 6 School of Medical Sciences, Edith Cowan University, Joondalup, Perth, WA 6027, Australia

## Abstract

Regulation of tissue development and repair depends on communication between neighbouring cells. Recent advances in cell micro-contact printing and microfluidics have facilitated the *in-vitro* study of homotypic and heterotypic cell-cell interaction. Nonetheless, these techniques are still complicated to perform and as a result, are seldom used by biologists. We report here development of a temporarily sealed microfluidic stamping device which utilizes a novel valve design for patterning two adherent cell lines with well-defined interlacing configurations to study cell-cell interactions. We demonstrate post-stamping cell viability of >95%, the stamping of multiple adherent cell types, and the ability to control the seeded cell density. We also show viability, proliferation and migration of cultured cells, enabling analysis of co-culture boundary conditions on cell fate. We also developed an *in-vitro* model of endothelial and cardiac stem cell interactions, which are thought to regulate coronary repair after myocardial injury. The stamp is fabricated using microfabrication techniques, is operated with a lab pipettor and uses very low reagent volumes of 20 μl with cell injection efficiency of >70%. This easy-to-use device provides a general strategy for micro-patterning of multiple cell types and will be important for studying cell-cell interactions in a multitude of applications.

The emergence of microfluidic organ-on-a-chip systems and the ongoing efforts to mimic live organ physiology on a smaller scale led to renewed interest in the optimal conditions needed to support a cell’s culture in an artificially designed microenvironment^1^,^2^,^3^. The sub-micrometer feature resolution and accurate geometries that can be readily manufactured using soft lithography opened new frontiers towards the identification of optimal conditions to support such conditions^4^,^5^.

These advances can be used to study cell-cell modulation in organ formation and the *in-vitro* reconstruction of tissues for tissue replacement. For example, the interaction between stem cells and their niche regulate tissue regeneration^6^, co-culturing of HUVEC and fibroblasts assist in functional capillary formation^7^ and activated stromal fibroblasts assist in cancer initiation and progression^8^,^9^,^10^. These findings further stimulated a search for new methods to easily characterize the complex interactions between various cell types *in-vitro*; the simple form of which is 2D cell patterning.

Cell patterning approaches are common and broadly categorized into active versus passive patterning. Active patterning involves the application of an external force such as optical^11^, acoustic^12^,^13^,^14^ or fluidic^15^,^16^,^17^ forces to confine or transport cells to desired spatial positions. While they offer an on-demand capability, they generally require a skilled operator, complicated chip fabrication and specialized peripheral equipment such as lasers, pumps and waveform generators. More importantly, they are not well adjusted to the patterning of more than one cell type due to the incremental differences in the physical properties of different cell types.

Alternatively, passive patterning methods rely on the pre-stamping of a substrate with proteins or other hydrophilic chemical groups, followed by cell seeding and their selective attachment to the stamped regions^18^,^19^. In such methods the number of stamped molecules cannot be accurately controlled due to poor stamping repeatability^20^. Moreover, patterning of two cell-types using passive patterning is highly challenging as it requires the pre-patterning of two cell-selective molecules, as well as aligning the pattern between subsequent steps while eliminating the cell-to-cell cross-contamination^21^,^22^. Commercially available nano-drop printing system can solve some of these problems by depositing accurate nanoliter volumes of proteins in a high spatial resolution^23^. However, this method has its own drawbacks, such as high costs and low throughput.

Stencil-based patterning approaches have shown great promise for removing technical barriers required for engineering the cellular microenvironment^24^. In this approach, elastomeric stencils (i.e., made from Silicon, PDMS or parylene) can be used to pattern cells or biomolecules to specific regions of a substrate^25^,^26^. This approach allows easy control of the patterned cell density by directly adjusting the injected cell suspension concentration. However, the attachment of stencil over the substrates and residual of the cells or biomolecules that remain on non-perforated areas can cause trouble in large-scale applications. Cell patterning using switchable surfaces that can be turned from being cell repulsive to adhesive by specific stimuli such as electrical potential or temperature has been also demonstrated^27^,^28^. Nonetheless, these approaches are still complicated to perform and as a result, are seldom used by biologists.

A platform that circumvents most of these problems in patterning cells in an open configuration using spatial confinement was presented by Chiu *et al*. using multilayer soft lithography^29^. In spite of its multiple advantages, its fabrication involves complicated multilayer soft lithography and is operated by external pump, which limits its widespread adoption. Therefore, although desirable, to date there is no simple method to easily stamp two types of adherent cells in a desired pattern on a 2D substrate.

We describe here a new microfluidic stamp which utilizes a novel valve design to rapidly and accurately pattern two arbitrary live adherent cell types on a flat substrate in an interlaced configuration. The method does not require multilayer device fabrication; it is compatible with conventional soft-lithography and can be operated with a standard hand-held pipette by non-expert personnel.

## Material and Methods

### Device design and fabrication

**Figure 1:**
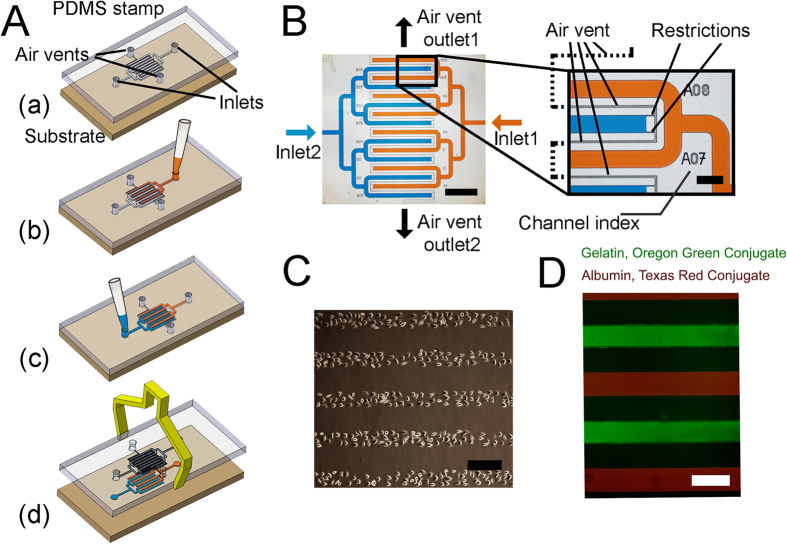
Microfluidic stamp structure and patterning. (**A**) Stamping procedure: (a) The stamp is placed on a flat substrate such as a glass or a tissue-culture dish. (b) Cell suspension is injected to the first inlet using a standard lab pipettor. The cell suspension fills the channels while allowing residual air to escape through the air vent. (c) In a similar manner, a second cell suspension is injected from a second inlet. (d) Following cell sedimentation and on-chip incubation, the PDMS stamp is removed while leaving cells attached to the substrate. (**B**) Device channel visualization by showing image of the device injected with blue and orange food colours. Zoomed image shows the restriction and air vent that limit liquid flow but allow air to escape. Scale bar, 1 mm and 200 μm (**C**) Fibroblasts L929 patterned on a TC plate. Scale bar 300 μm (**D**) Fluorescent-conjugated protein patterning. Scale bar: 500 μm.

In this work, we introduce a simple method for creating protein and cell patterns on a variety of substrates using a novel microfluidic stamp. The device design and its operation principle are depicted in Fig. 1A. In this device, a liquid, such as cell medium, is loaded into the main channel that is sequentially branched to multiple dead-end microchannels. Air freely passes through narrow restrictions positioned at the end of each channel, while the liquid stops due to Laplace pressure. Unlike common microfluidic devices where the PDMS slab is hermetically sealed against a substrate using plasma bonding, here the PDMS stamp is temporarily attached to a substrate, thus facilitating cell loading without any leakage. Assuming that (1) the contact angle between the liquid and PDMS walls is constant (2) the channel’s depth is much smaller than its width and (3) the channel’s cross section and the restrictions’ cross section are both uniform along the channel’s length, the failure-free injection pressure in the PDMS stamping device is determined only by the Laplace pressure caused by the air/liquid interface curvature at the restriction. Therefore, the allowed injection pressure depends only on the restriction width, regardless of channel dimensions. This makes manufacturing PDMS stamping device with different channel size possible as long as restriction width is well designed. In practice, the strength of these assumptions depends on the fabrication uniformity/quality and is complicated by the fact that there are two different contact angles: one between the liquid and the stamp PDMS walls, and another between the liquid and the cell-stamped substrate.

To fabricate PDMS stamps with a flat surface, we employed standard microfabrication techniques to make silicon master moulds using SOI (Silicon on Insulator) wafers with the following specifications: 100 mm wafer diameter, 80 ± 1 μm device layer, 2 μm buried oxide layer, 500 ± 15 μm handle layer. Photolithography on SOI wafers was performed using a Karl Suss MA6 Mask Aligner (SUSS MicroTec, Germany) followed by deep reactive ion etching (DRIE) using an STS system. The patterned SOI wafer was silanized with trichloro (1H, 1H, 2H, 2H-perfluorooctyl) silane (Sigma Aldrich, USA) to render the surface hydrophobic. PDMS prepolymer was prepared by mixing the PDMS at a standard 1:10 ratio (Sylgard 184, Dow Corning, USA) and degassing in a vacuum chamber for 2 hr. PDMS mixture was poured onto the SOI mould and cured at 80 °C for 2 hr inside a conventional oven. The PDMS was then cut from the mould, and fluidic access holes were punched into the device. All the devices were washed with isopropanol and DI water and then dried using N_2_ gas. Prior to use, all the PDMS stamps were autoclaved at ~120 °C for 20 min. The fabricated stamps with the desired patterns were brought in conformal contact with the substrate and, if necessary, pressed together to create a seal.

### Cell isolation and culture

Co-culture experiments were conducted with primary cardiac-derived mesenchymal stem cells and mouse aortic vascular endothelial cells with a 1:1 ratio mixture of each cell’s specific medium. Both cell lines where isolated and expanded by the Victor Chang Cardiac Research Institute (Sydney, Australia). Cardiac-derived mesenchymal stem cells are a rare subset of cells isolated from the interstitial fraction of adult murine heart by FACS using SCA1+/PDGFRα+/PECAM- marker expression^30^. To facilitate live cell imaging using green fluorescent protein expression, cells were isolated from a transgenic mouse that expresses ‘enhanced’ GFP (EGFP) under the control of the chicken beta-actin promoter^31^. The complete procedure for isolation of cardiac mesenchymal stem cells is reported in supplementary document (see Method S1). Isolated cells were then plated at a cell density of 5,000 cells in 35 mm culture plates with complete culture medium of αMEM, 20% FBS (v/v), 100 μg/ml of Penicillin, 250 ng/ml of Streptomycin and 200 mM of L-Glutamine. Plated cells were incubated at 37 °C under 5% CO_2_ in a humidified incubator. Fresh culture medium was changed following the initial day of isolation and on every third to fourth day thereafter. Prior to replenishing the media the cells were washed twice with sterile PBS to remove non-adherent cells and debris and the cells were treated with TrypLE (Life Technologies, Carlsbad, CA).

Mouse aortic vascular endothelial cells were isolated from the p53-deficient mouse aorta and cultured in M199 medium (Invitrogen) with 5 ng/ml of recombinant vascular endothelial growth factor (VEGF; Sigma-Aldrich), 5 ng/ml Hepes (Invitrogen), 10 mM heparin sodium (Sigma-Aldrich) and 5% FBS. Mouse aortic vascular endothelial cells from p53-deficient mouse were cultured for over 100 passages as described elsewhere^32^. For the sake of simplicity, we will hereafter refer to cardiac-derived mesenchymal stem cells as SC and to mouse aortic vascular endothelial cells as EC.

### Fibroblast cell culture and cell viability assay

Fibroblast L929 cells (Sigma Aldrich, 04102001-1VL) were cultured using 10% serum, 1% PS in DMEM in a T-75 flask and the medium was changed every 3 days. The cells were passaged at 80% confluency and cultured with a seeding density of 10^4^ cells/cm^2^. Calcein AM and Propidium Iodide (PI) was used for live and dead staining. Fibroblast cells were injected into the device using hand-held pipettor and were left to attach for 4 hr. The stamp was peeled-off from a 6-well plate and washed once prior to staining. The cells were incubated with 4 μM Calcein AM and 1 μM PI for 1 hr followed by medium replacement. Prior to imaging, the staining efficiency was checked using an Olympus IX73 fluorescent microscope.

### Protein stamping

Gelatin from Pig Skin, Oregon Green 488 Conjugate (G13186, 5 mg) and Albumin from Bovine Serum (BSA), Texas Red conjugate (A23017, 5 mg) were purchased from ThermoFisher scientific. Protein stock solutions of 1 mg/ml were prepared and stored in refrigeration and were protected from light to minimize photo-bleaching. Before stamping, a solution of Gelatin (0.2 mg/ml) and Albumin (0.4 mg/ml) in DI water were prepared by proper dilution. The microfluidic stamp was placed on a pre-washed glass slide and injected with the diluted protein solutions. Following an incubation of 1 hr at room temperature to increase protein adsorption to the glass slides, the stamp was peeled off and the glass slide was washed with DI water, dried with an air gun and fluorescently imaged with an Olympus IX 73 inverted fluorescent microscope.

### Microscopy

#### Fibroblast viability

Imaging of the attached fibroblast L929 cells was performed using the Olympus IX73 inverted microscope. At each time point both phase-contrast and fluorescent channels were captured. For each stamped area three arbitrary sections were tracked over time by marking the well plate bottom. The stamps were carefully peeled off the well plates using sterile (autoclaved) tweezers and covered with fresh medium. The bright field images of the attached cells were captured, while the two remaining plates were transferred back to the incubator (to be stained and imaged after 24 and 48 hr, respectively). Each of the devices was imaged before the staining to account for washed cells. Next, the devices were peeled off and the cells were stained. Following 30 min incubation, the TC plate was imaged using bright-field and fluorescent GFP/TXred channels.

#### Time lapse microscopy

Time lapse microscopy with an Olympus IX83 equipped with a dedicated CO_2_ incubator (37 °C) was performed to track cell migration in the SC-EC co-culture experiments as well as assessment of Fibroblast cells proliferation. Microphotographs of a defined region of the device were taken with 20 min intervals for 6 consecutive days. The defined region includes 6 regions each on a separate stamping area where the quality of the images was adjusted individually. To avoid cell drifting, the stage speed was reduced to 1 mm/sec.

### Image and data analysis

#### Collective cell migration analysis using ImageJ

Tracking analysis of time-lapse microphotographs was performed using the manual tracking ImageJ software plug-in (NIH, Bethesda, USA). The generated tracking data were used to graphically plot the trajectories of the SCs and ECs and to calculate the motility data. SCs and ECs were counted by segmentation using the MATLAB Image Processing Toolbox as described in the supplementary document (see Method S3).

#### Viability and proliferation analysis

Analysis of viability and proliferation was done by counting the number of cells in specific images using ImageJ software. The threshold was optimised for each image to achieve separated and distinguishable cells with no background noise. The particle analysis tool was used to count the number of cells in each image.

#### Fibroblast L929 microfluidic culture to measure proliferation

Fibroblast L929 cells were cultured in Dulbecco’s Modified Eagle Medium (Invitrogen) under humidified condition (5% CO_2_ at 37 °C) at concentration of 10^6^ cells/ml. The cells were passaged every 3 days at 80% confluency. The PDMS chips were carefully cleaned using distilled water and scotch tape and inspected under a microscope to assure complete removal of dust particles. All the instruments required for the experiment including the 100 μl and 1000 μl pipet tips, the tweezers and 6 PDMS devices were autoclaved and then dried in oven for 2 hr. The devices were then placed inside a 6 well plate using the tweezers and pressed gently to ensure appropriate contact is established between the chip and the substrate. Cell suspension of 4 × 10^6^ cells/ml was prepared using fibroblast L929 cells, harvested from two flasks. For each passage a Vi-CELL cell viability analyser (Beckman Coulter) was used to ensure viability of >90%. For each injection 20 μl of cell suspension was aspirated using a pipettor and injected into the device. Next, the cell concentration in all devices was checked under a microscope and 2 ml of DMEM medium was added to each well. The devices were then placed in the time lapse microscope and were tracked for 4 hr to ensure cell attachment. Upon attachment, the time lapse experiment was paused and the devices were peeled gently under sterile conditions. Subsequently, 1 ml of fresh medium was added to each device to cover the patterned cells, the well plate was placed back to its original position under the microscope and the microscope operation was resumed. Recorded movies before and after device detachment was checked for proper overlapping.

#### CMSCs-MAEC microfluidic co-culture

For the co-culture experiments, one T-75 confluent flask of SCs and one T-75 confluent flask of EC were used and a cell suspension with concentration of 4 × 10^6^ cells/ml was prepared for each of the cell types. Next, a co-culture device was placed inside a single well of a multi-well plate, while assuring that it is properly sealed against the well’s substrate. A 20 μl of the EC cell suspension was injected into the first inlet of the co-culture device. Next, a 20 μl of the SC cell suspension was injected to the second device inlet. After an incubation of 4 hr, the cell attachment was verified based on cell morphology changes. Following successful cells attachment, the microscope was paused and the devices were peeled using sterile tweezers. A 1 ml mixture of SC and EC medium (1:1 ratio) was added to each well to cover the exposed cell pattern and the plate was placed back to its original position at the microscope stage. The time step and total experimental time were set to 30 min and 7 days, correspondingly.

## Results and Discussion

### Device design and operation

#### Cell Stamping

In a standard micro-contact printing, in order to avoid cross-contamination, proteins normally stamped on a hydrophobic substrate to prevent non-specific cell attachment. This normally causes an undesirable confinement of the stamped cells to the protein-patterned region, suppresses cell motility and therefore limits cell migration studies. The described co-culture stamping method in this study can overcome this limitation by direct cell seeding on a treatment-free substrate, allowing unrestricted post-stamping cell migration.

The stamp operates in four steps, as shown in Fig. 1. Initially, a cured PDMS slab is cut from the master mould, followed by punching of two holes for the inlets and two holes for the air vents. Unlike common devices where the PDMS slab is hermetically sealed against substrate using Oxygen plasma, here the PDMS slab (stamp) is placed against a flat substrate, such as a polystyrene TC dish or a glass slide. In spite of the fact that the sealing is reversible, the stamp flatness ensures that the injected liquid is confined between the channels and does not leak. A cell suspension is injected using a standard lab pipettor to the first inlet. The solution flows and fills the channels till the liquid/air interface reaches the end of each channel. There, it encounters a restriction that exhausts air through an air vent while limiting the progression of the liquid due to Laplace Law. The same procedure is repeated with a second cell suspension, which is injected from an opposing inlet, thus forming an interlaced configuration of alternating solutions. Following rapid cell sedimentation, the stamp/substrate assembly is incubated for 4 hr until cells adhere to the substrate. Eventually, the stamp can be detached from the substrate and the patterned cells are covered with fresh medium to sustain their viability.

#### Protein stamping

In case cell migration is less of a concern, hydrophilic protein stamping using micro-contact printing can be used to pattern cells to desired regions on a substrate by selective cell attachment to hydrophilic protein moieties. Nevertheless, the ability of patterning two different protein patches in close proximity remains challenging due to the difficulty to maintain proper alignment of multiple stamping steps. The presented co-culture stamping method in this study overcomes this problem by injecting two different protein solutions to the two inlets as shown in Fig. 1D followed by a short incubation at room temperature, peeling and washing of the substrate. To demonstrate the feasibility of such a dual-protein stamping, fluorescently-conjugated proteins, Gelatin (Oregon Green) and Albumin (Texas Red) were selected. Similar to the cell stamping, adjustment of the protein concentration injected to each of the channels allows controlling the number of protein molecules adsorbed to the substrate.

### Device characterization

#### Stamp design, fabrication and leakage testing

**Figure 2:**
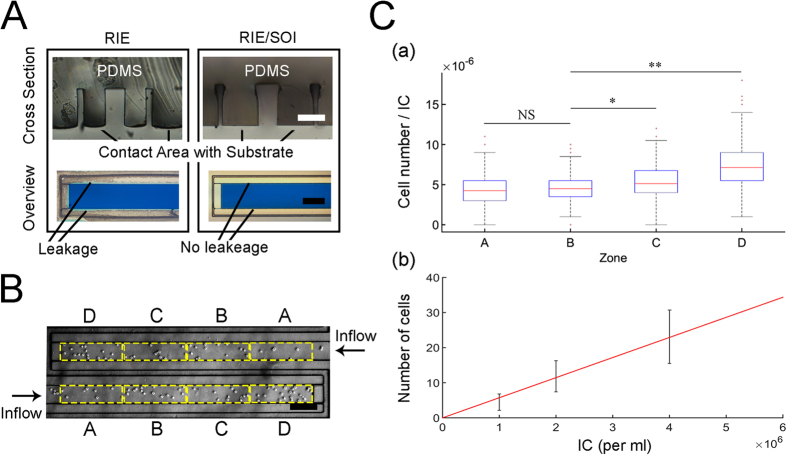
Device Characterization. (**A**) Comparing SOI (Silicone-On-Insulator) vs. Non-SOI wafer fabrication and the resultant leakage-proof performance. Scale bar 50 μm (**B**) Characterization of cell uniformity distribution in the channels by injecting L929 fibroblasts. Each channel was divided to four zones (**A–D**) which were indexed based on the directionality of the flow. Scale bar, 200 μm (**C**) (a) Boxplot of the normalized cell number in each of the zones for the injected bulk cell concentrations of 10^6^ cells/ml, 2 × 10^6^ cells/ml and 4 × 10^6^ cells/ml (b) Linear curve fit between average number of cells in each zone and the inoculum concentration (cell suspension concentration).

The restriction at the end of each stamp’s channel limits the flow of liquid, but allows air to flow out of the device. To improve the stamp’s performance the restriction width should be minimized. Although the device can be fabricated with SU8 photoresist, the limited aspect ratio of the resultant restriction would limit the range of operating pressures. DRIE was therefore used as a means to achieve better aspect ratio as high as 1:8. Figure 2A shows successful stamp loading. It also demonstrates that the anisotropic RIE fabrication using a standard silicon wafer results in compromised stamp flatness, as shown in the PDMS cross section, which led to leakage of liquid and its accumulation between the PDMS stamp and the substrate. In order to avoid this leakage, the stamp’s contact area with the substrate needs to be flat. To achieve that, together with restrictions of high resolution, a mask was patterned directly on a SOI wafer, followed by DRIE etching through a device thickness of 80 μm till the buried oxide layer was reached. For simplicity we used a device thickness of 80 μm in all of our experiments. This assured that the restriction’s width could be reduced down to 10 μm and also supports leakage-free operation due to high degree of stamp flatness. The high tolerance of device thickness also translates to repeatable calculation of the injected liquid and therefore allows the user to control the patterned cell density. The range of operating pressure of the device is provided in Table S1.

#### Cell distribution uniformity

One of the desired characteristics of cell patterning is to achieve a uniform cell distribution (per area). To characterize the cell distribution uniformity along the channels, fibroblasts L929 were injected at three volumetric cell concentrations of 10^6^ cells/ml, 2 × 10^6^ cells/ml and 4 × 10^6^ cells/ml. The resulted cell distribution along the channels was measured by counting the number of cells per quadrant, as shown in Fig. 2B. Following this approach we characterized the cell distribution in each zone, as shown in Fig. 2C by calculating the number of cells per quadrant normalized by cell suspension concentration (Inoculum concentration; IC) for three injected cell concentrations. To calculate the average number of cells per zone one can simply multiply the normalized number of cells per quadrant by the concentration of the cell suspension. As seen from the boxplot, higher cell concentration was found at the distal side of each channel, compared with the proximal zones. To verify it we performed a single factor ANOVA test and found a significant difference between the four zones. Next, we performed a t-test between each two zones and found a significant difference (*p* < 0.05) between the distribution of cells at zone A and zone B compared to zone C and zone D, as shown in Fig. 2C. The difference is the result of rapid sedimentation of cells at the tip of the pipettor right before injection. This effect could therefore be reduced by either minimizing the time till injection is initiated, or with design of a longer channel while discarding the channel end. Figure 2C-b also demonstrates a linear relationship between the average number of cells and the corresponding cell suspension concentration.

It was found that near the inlets there is increased cell sedimentation due to flow perturbations. As a result, injection efficiency to the device was lower than 100%, where injection efficiency is defined as the observed cell concentration in the channels divided by the nominal bulk cell concentration before injection. The observed cell concentration was calculated by counting the number of cells in each channel (while excluding sedimented cells at the vicinity of the device inlet) and dividing it by the channel’s volume (22.8 nl). The bulk cell concentration was measured by Vi-CELL cell viability analyser. It was found that the average injection efficiency for cell concentration in the range of 1 × 10^6^ cells/ml to 4 × 10^6^ cells/ml was ξ = 83% ± 10%. Therefore, the seeded cell density per area is adjusted by calibrating the injected bulk cell suspension concentration, which could further be selected independently for each of the two cell channels. The cell density per area is then given by *D* = *ξ. ρ. h* where *D* is the cell density per area in the channels, *ρ* is the injected bulk cell density, *h* is the stamp depth and *ξ* is the cell injection efficiency. As mentioned before, due to the fabrication method (SOI wafer), the stamp thickness *h* has a high accuracy of down to the few micrometers. Using a uniform and accurate stamp thickness therefore results in increased accuracy of the patterned cells density (per area).

#### Cell viability and proliferation

**Figure 3:**
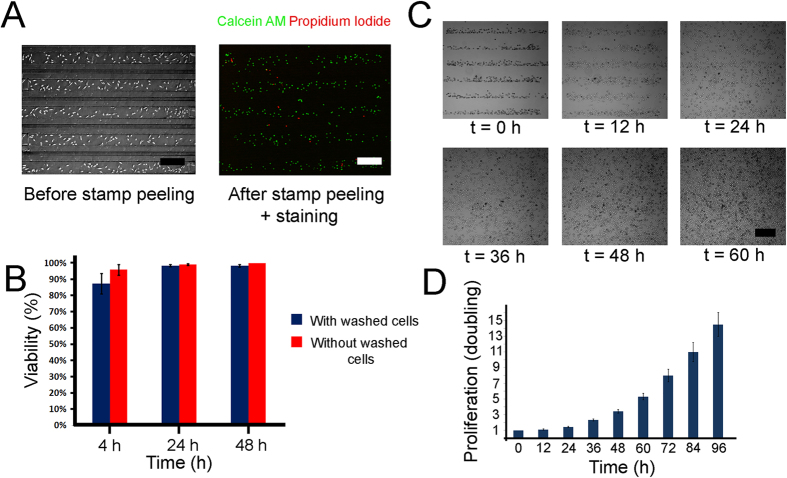
Post-stamping cell viability and proliferation. (**A**) Image of Fibroblast L929 Cell in stamp and after stamping peeling + Calcein/PI viability staining. (**B**) Quantification of post-stamp cell viability at t = 4 hr, 24 hr and 48 hr. Viability was measured for both the case of counting/ignoring post-peeling washed cells. (**C**) Images of fibroblast proliferation over the course of 60 hours. Here t = 0 h is the time when the stamp was peeled. (**D**) Fibroblast proliferation (fold expansion), after stamp is peeled-off (t = 0 hr) tracked over the course of 96 hours.

Following the stamp characterization we checked the cell viability and proliferation. The post-peeling cell viability is important to assure that the peeling process did not compromise normal cell functionality or inadvertently caused rapid cell death. In addition, it is important to verify that the cell functionality remains unperturbed before and after the cell injection. Ideally, the desired cell proliferation and spreading should not depend on a specific pattern. There are some challenges associated with cell culturing in microfluidic devices including nutrient depletion and insufficient gas exchange occurring due to their small culturing volume. In our device, the cell culture surface and volume are 0.92 mm^2^ and 54 nl, respectively for each channel branch (corresponding to surface-to-volume ratio of 17) which is within the recommended range suggested by Halldorsson *et al*.^33^. To characterize viability and proliferation we injected Fibroblasts L929 to the device, while sealing it against a TC dish. Following a 4 hr of incubation, the stamp was peeled off, the cells were covered with fresh medium and were double stained with a live/dead staining kit (see Method S2), as shown in Fig. 3A. During the stamp peeling and medium coverage, a small fraction of the cells were washed. Since the number of washed cells was small it was not possible to estimate their viability using conventional methods. We therefore calculated the viability twice. Once while assuming the washed cells as dead and another while ignoring the washed cells, as shown in Fig. 3B. It can be seen that cell viability remains >85% in all experiments.

The functionality of the cultured cells in terms of proliferation rate was also investigated using time-lapse microscopy. We patterned L929 Fibroblasts and counted their doubling versus time over a period of 4 days. As shown in Fig. 3C and unlike typical protein-based stamps which cause biased cell confinement, in the current stamping method, the cells freely migrate across the substrate and are not limited to the patterned region. Interestingly, after 24 hr incubation the original cell pattern is barely discerned, confirming the suitability of this technique for long-term cell co-culture. To quantify the cell proliferation the cell doubling was tracked over a period of 96 hr, with intervals of 12 hr. As shown in the Fig. 3D there is high proliferation rate with a doubling time of ~24 hr, comparable with typical cell seeding at these densities.

#### Interaction of cardiac-derived mesenchymal SC and EC in patterned co-culture

It is technically challenging to study the dynamics of cell-cell interactions *in-vivo* by single cell fate mapping. The co-culture stamping device allows one to model these interactions in-vitro. One isolates two well-defined cell types while tracking their individual fates by live cell imaging. Such an *in-vitro* co-culture assay can be used to study the signalling and development pathways that may occur *in-vivo*. To demonstrate this concept we isolated a subpopulation of cardiac interstitial cells from adult mouse hearts which contribute to the perivascular niche controlling vascular development and responses to injury. These cells form colonies when grown in tissue culture, have characteristics of mesenchymal stem cells with specific *in-vitro* and *in-vivo* properties related to their epicardiac origin^30^. It is hypothesised that cardiac-derived mesenchymal SCs secrete growth factors that direct tissue repair after myocardial infarction (MI), including revascularisation of the infarct region after dead cardiomyocytes are removed by phagocytic cells. Sprouting angiogenesis into the infarct zone may be driven by cardiac mesenchymal SCs which reside there early on after MI. Therefore, the migratory and proliferative behaviour of cardiac mesenchymal SCs and ECs in patterned co-culture was studied by time lapse microscopy.

**Figure 4:**
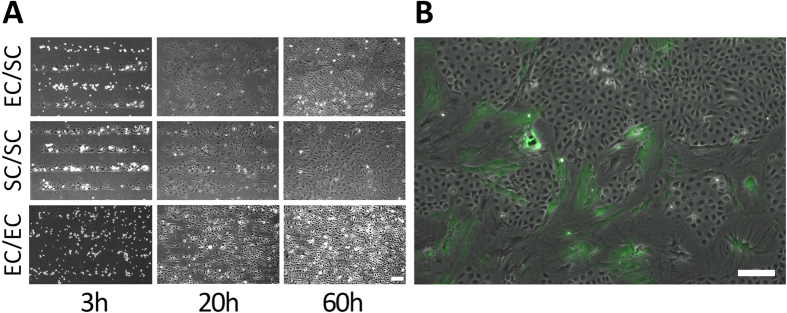
Endothelial/Cardiac Stem Cells co-culture. (**A**) Selected time lapse images of cardiac stem cells (SC) co-cultured with Endothelial Cells (EC) at time t = 3 h, 20 h, 60 h and their corresponding single-stamp culture of Endothelial Cells only (EC/EC) and Cardiac Stem Cells only (SC/SC). Scale bar: 200 μm. (**B**) Overgrowth of Cardiac Stem cell clusters by neighbouring Endothelial cells in a Co-culture experiment. Scale bar: 200 μm.

Figure 4A shows a sequence of images of the co-culture stamping (EC/SC) at three different time points, accompanied by controls that include a single cell culturing of either stem cells (SC) or endothelial cells (EC). As shown in the figure, the stem cells proliferate at a low rate and similarly to fibroblasts gradually migrate away from their original stamping position (See Movie S1). In parallel the EC proliferate at a much faster rate and, when they reach the stem cells they confine them to narrow filaments, as shown in Fig. 4B. This confinement is observed only in the co-culture experiment and is absent from the two single-culture controls (See Movie S1 and Movie S2).

**Figure 5:**
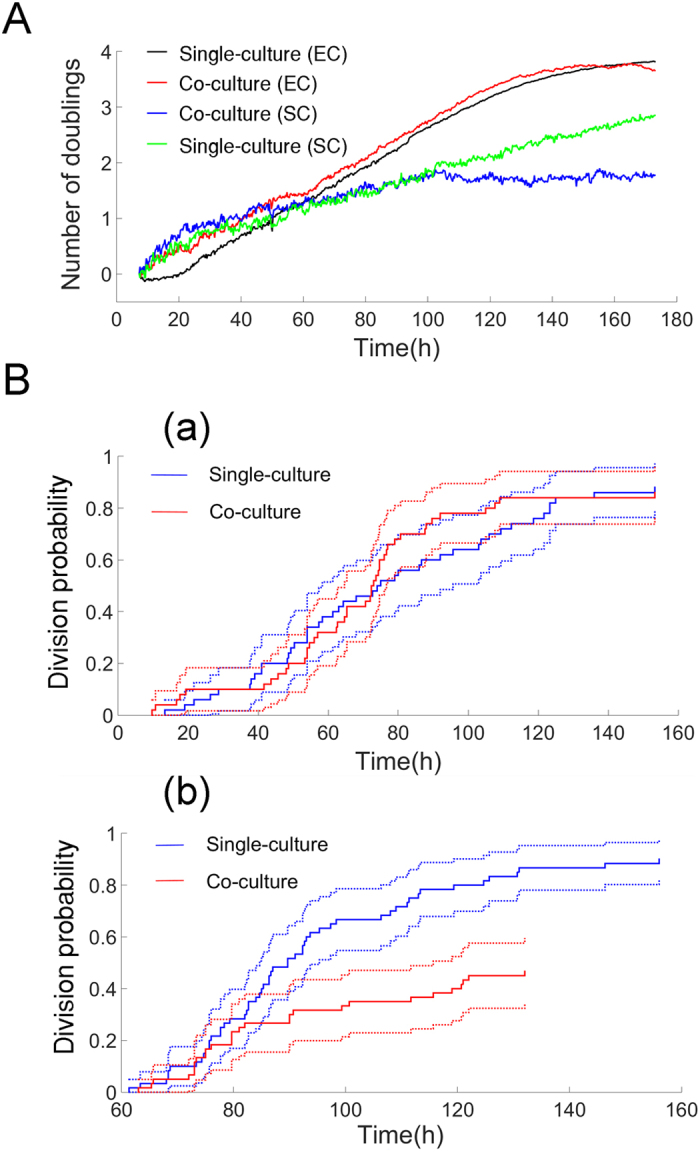
(**A**) Proliferation of Endothelial cells and Stem cells in the single-culture versus co-culture reported as number of doublings versus time. (**B**) Kaplan-Meier and Cox regression analysis of time to division of SC in single-culture (SC|SC) versus co-culture (SC|EC) (a) Probability of division measured from the beginning of the experiment (b) Probability of division starting at t = 60 hours (risk factor = 0.34 ± 0.09 and p = 5.53 × 10^−6^).

To investigate the effect of co-culture on each cell type’s growth, proliferation of SC and EC was quantified using MATLAB Image Processing Toolbox (See Methods S3). The proliferation rates of SC or EC were estimated by segmenting the number of EC or SC when stamped in co-culture (EC|SC) or in single culture as a control (EC|EC or SC|SC, respectively). The time course of EC and SC proliferation expressed as the number of doublings – log_2_ [cell number] – is shown in Fig. 5A. A linear regression model was fitted to the data (n = 3 movies per condition), showing the effect of co-culture vs single culture and cell type on growth lag (intercept) and growth rate (slope). The doubling times for the single culture of EC (black curve) and single culture of SC (green curve) were 40.0 ± 0.2 hr versus 55.0 ± 0.2 hr, respectively (p < 0.001). The growth curve of EC (control) was sigmoidal with a 10 hour growth lag. The growth lag of EC was lost when grown with SC (red curve, p < 0.001). Thus SC provides a microenvironment that increases the initial growth of EC. In contrast, co-culture of SC with EC resulted in growth arrest of SC after 100 hours (blue line, p < 0.001).

Kaplan-Meier and Cox regression analysis was performed to compare effect of co-culture or single culture on the probability of stem cell division. Cells which did not divide during the course of the study were considered to be right censored. As seen in Fig. 5B-a, there was no effect of co-culture on the probability of stem cell division initially; however after 60 hours, co-culture reduced the probability of stem cell division (Fig. 5B-b, relative risk = 0.34 ± 0.09, p = 5.53 × 10^−6^). This means that the relative risk of division of SC in the co-culture is around 1/3 of the risk of division in single culture. These observations are consistent with growth arrest of SC in co-culture at 100 hours (Fig. 5A, blue curve).

**Figure 6:**
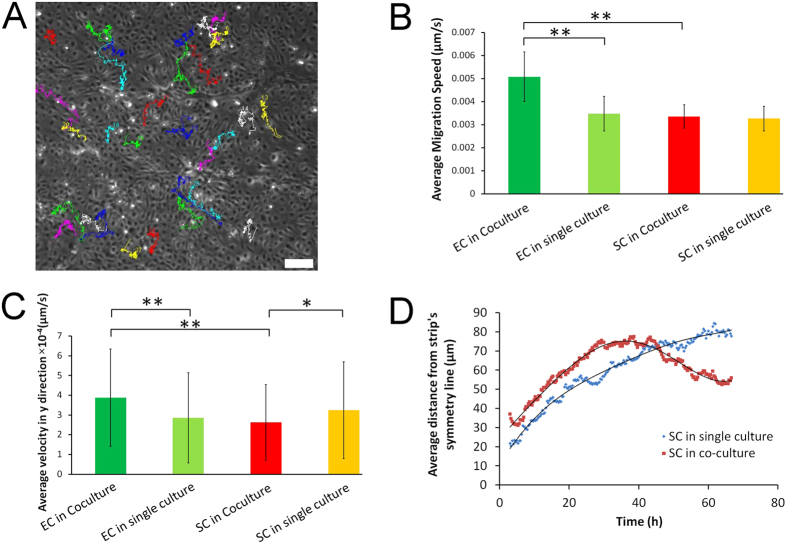
Single-cell tracking and collective cell migration analysis (**A**) The trajectory of multiple Endothelial Cells (EC) in the co-culture (**B**) Average migration speed of EC and SC in single culture and co-culture (n = 30 for each condition) Scale bar: 200 μm. (**C**) Average migration velocity normal to channel direction for EC and SC in single-culture versus co-culture (n = 100) (**D**) Variation of the relative distance of Cardiac Stem Cells from the symmetry line of their corresponding strip with time in the co-culture vs. single-culture.

To quantify the collective cell migration we used ImageJ to track the trajectory of single cells. Figure 6A shows the colour-indexed tracking of 30 cells along axes normal to the channels. As we can see, tracked cells show different migratory behaviour. Some of the cells move faster, some of them show migration in a definite direction (See Movie S3). Therefore, in order to distinguish between different migratory patterns, we defined “cell type” and “culture conditions” as fixed conditions and reported “migration speed”, “average migration velocity normal to the channels” and “Forward Migration Index (FMI) normal to the channels” as their function.

Figure 6B presents the average migration speed of cells of ECs and SCs under single- or co-culture condition. ECs have higher average speed in comparison to SCs. In addition average migration speed of ECs was higher in co-culture compared to single culture (p < 0.01).

In order to assess the directional migration of both cell types, the average migration velocity normal to the channels was calculated for 100 cells of each type as shown in Fig. 6C and three statistically different groups were found. Interestingly, the average migration velocity of EC in the co-culture (mean value = 3.87 × 10^−4^ μm/s) was higher than the single culture (mean value = 2.86 × 10^−4^ μm/s, p < 0.01) while SC showed higher values in single culture compared to co-culture (p < 0.05). This was the results of a difference in collective cell migration of EC and SC and its inhibition as cells encounter adjacent patterns. Figure S1 demonstrates the histogram of average migration velocity of EC normal to channels. As shown in this figure, in case of single culture, the distribution is skewed to the left with a high frequency of cells with low y-velocity.

In the case of the SC, as observed from Fig. 6D, the cells reverse their migration direction when they are in the co-culture due to EC confinement. To account for this direction change we tracked the distance of SC from the symmetry axis of each stripe, while each cell is measured relative to the axis of the pattern it originated in. As can be seen from the trajectory of multiple cells in Fig. 6D, the SC initially migrates away from the symmetry axis, followed by reversing their direction. This is because EC overgrow SC, and displace SC back to the symmetry axis to form the observed elongated bundles. In contrast, in the case of the single culture controls, the cells move away from the symmetry axis of each pattern with symmetric migration rates without a change in direction.

During collective cell migration as well as single cell migration, cells may move in straight lines or move around randomly. Therefore, the behaviour of cells of different types in a specific population can be quantified by defining directional migration of that type. Figure S2 shows the forward migration index (FMI) of EC and SC cells under different culture conditions in y-direction. FMI is defined as the displacement of a cell in a certain direction (e.g. y-direction) divided by the total path length of that cell^34^. As presented in Fig. S2, EC cells show higher FMI compared to SC (p < 0.05) in both culture conditions. The FMI plot confirms that the directional migration of cells in the y-direction depends on the cell type regardless of the culture conditions.

## Conclusions

To explore cell homotypic/heterotypic interaction we present here a new stamping method to rapidly pattern, in close proximity, two arbitrary adherent cell types in an interlaced configuration of choice. Following the stamp peeling, the two cell types are free to migrate across the stamped substrate and interact to form a tight interlaced 2D complex cell structure. The stamp design is based on a novel in-built valve which restricts the injected liquid flow while allowing residual air to vent out of a shared central channel.

SOI-based RIE etching was used in order to achieve a defined device depth and improved channel cross section uniformity. Once a master mould is ready, the number of manufactured stamps is not limited. Furthermore, a single PDMS stamp can be reused multiple times as long as the inner stamp’s surface hydrophobicity is not compromised due to protein adsorption.

One limitation of the presented method is its incompatibility with a more realistic native 3D cell patterning configuration. Nevertheless, this limitation is shared by the majority of the existing cell patterning techniques and is therefore less of a concern. In addition, 3D cell patterning requires more specialized imaging techniques, such as confocal imaging, restricting their widespread usage.

Using our stamping device, we investigated the collective migration of EC/SC by measuring the average migration speed, average migration velocity in y-direction and cell directionality based on two fixed parameters of cell type and culture type. Statistical analysis of our data revealed that ECs in the co-culture are more motile and migrate faster in the direction normal to channels compared to single culture. Moreover, looking at the migration measurements of EC and SC in the co-culture condition, ECs were more motile and ran faster in the direction normal to the channels compared to SCs. Analysis of the proliferation rate of each cell type revealed that SC provides a microenvironment that increases the initial growth of EC. In contrast, co-culture of SC with EC resulted in growth arrest of SC after 100 hours. The Kaplan-Meier estimate of time to division and Cox regression analysis demonstrated that after 60 hours, the relative risk of division of SC in the co-culture was reduced. Growth arrested SCs differentiated into cells with myofibroblast or spindle shaped morphology (Fig. 4b). Finally, migration of SC away from symmetric axis of their seeding strip was reversed by EC from the neighbour seeding strip, which had a greater proliferation rate, invading and undermining GFP positive differentiated SC.

We believe that the presented stamping method may open up new opportunities to control patterned cells’ microenvironment. We envision that the proposed device can be used to generate a hierarchical multilayer protein layers by repeated stamping steps, where each layer can harbor two different protein types/concentrations. This bottom-up effect can further be complemented by a top-down effect, where the patterned cells are incubated with various biomolecules such as growth factors, cytokines, drugs or a combination of their concentrations. The freedom to engineer the cells’ microenvironment, together with the preservation of their motile capability can offer valuable information on multi-cellular migration behaviour under external stimuli.

The described method can be used to engineer a more complex cell patterning configurations by adding new channels in a triple (or more) interlaced configuration, subject to geometric limitations that allow air to be vented out during the liquid filling stage. Therefore, there is no limitation on the number of patterned cells in the device. Furthermore, the presented stamping method can be considered as a candidate for a new approach towards complex three-dimensional organ printing. This could be achieved by repeated stacked stamping of gel-encapsulated cells. Using such printing strategy could lead to the design of complex interlaced 3D sheets or semi-tubular geometries. A layer-by-layer cell printing, in contrast to pixel-by-pixel cell printing could offer higher throughput and the elimination of an in-plane printing misalignment.

To conclude a novel leakage-free stamping was developed to pattern adherent cell lines and to study the interaction of endothelial and cardiac stem cells by live cell imaging, quantifying the effect of co-culture on cell proliferation and migration. The stamp is fabricated using RIE etching and standard soft lithography techniques, does not require dedicated plasma bonding, and is operated with a standard lab pipette. To the best of our knowledge this is the simplest 2D microfluidic co-culture stamping device to date. We therefore believe it can be easily adopted by common non-microfluidic cell biology labs for the study of cell-cell modulation and paracrine signalling.

## Additional Information

**How to cite this article**: Hassanzadeh-Barforoushi, A. *et al*. A rapid co-culture stamping device for studying intercellular communication. *Sci. Rep.* **6**, 35618; doi: 10.1038/srep35618 (2016).

## Supplementary Material

Supplementary Information

Supplementary Movie S1

Supplementary Movie S2

Supplementary Movie S3
