# Supplementary material for: A rapid co-culture stamping device for studying intercellular communication
Source: Sci Rep. 2016 Oct 18;6:35618. doi: 10.1038/srep35618 (PMC5067516; doi:10.1038/srep35618)
Supplement: Supplementary Information [file srep35618-s1.pdf]

A rapid co-culture stamping device for studying intercellular communication

Amin Hassanzadeh-Barforoushi, Jonathan Shemesh, Nona Farbehi, Mohsen Asadnia,

Guan Heng Yeoh, Richard P Harvey, Robert E Nordon, and Majid Ebrahimi Warkiani

Supplementary information

Figure S1.

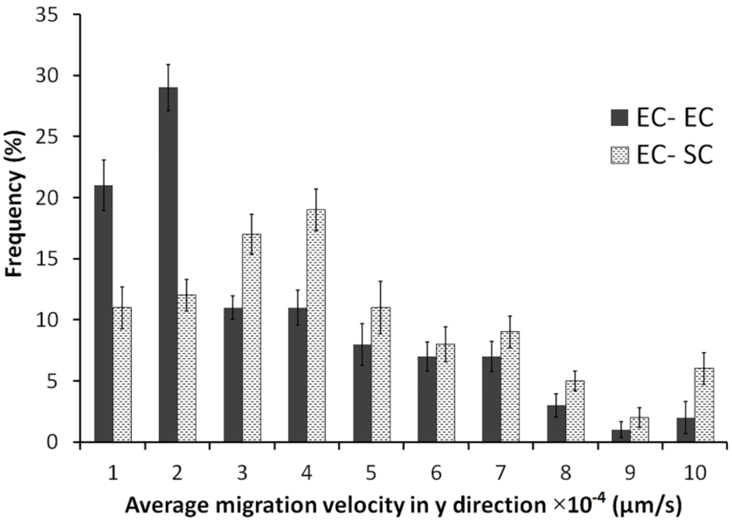

Average migration velocity distribution of Endothelial cells in single-culture versus co-culture (n=30).

Figure S2.

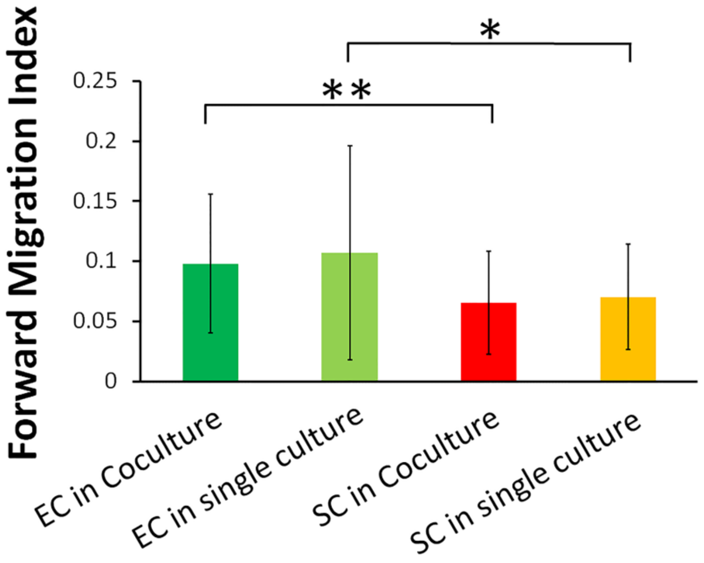

Forward migration index (FMI) in the y-direction for EC and SC in single-culture and co-culture (n=30).

SI Movie Legends

Movie S1
Video illustrating the migratory behaviour of Cardiac Mesenchymal Stem cells and Endothelial cells as well as formation of SC bundles over time. SCs are located on the rows with even row numbers and are clearly distinguished from endothelial cells based on their spindle shaped morphology (Scale bar= 200 µm).

Movies S2
Video illustrating the cord like structure formed by elongated SC cells in the SC-EC co-culture. Fluorescent SCs are in green (Scale bar= 200 µm).

Movie S3
Video illustrating the migration path of the endothelial cells in the SC-EC co-culture experiment. 30 cells (10 from 3 strips) are selected (Scale bar= 200 µm).

Method S1: Isolation of cardiac mesenchymal stem cells

Briefly, mice were sacrificed using cervical dislocation. The adult murine hearts were dissected while avoiding collection of cells from the outflow tract. After mechanical digestion of the hearts, the tissues were suspended in 5ml of 260 U/ml of pre-filtered collagenase Type 2 (w/v) (Worthington, Lakewood, NJ) in PBS and incubated in 37°C water bath. Every 6 min the sample was vigorously agitated. After 12 min the supernatant was collected in 3 mL of heat treated Fetal Bovine Serum (FBS) solution at 4°C. This process was repeated three times until the remaining heart tissue was completely digested and pooled in a

50 mL Falcon tube. The supernatant was then filtered using 40 µm filter strainers (Corning, 352340) and the cells were pelleted by centrifugation for 5 min at 600g. The supernatant was discarded and the cells were re-suspended in 1 mL of 2% FBS (v/v) in PBS. The cells were pelleted again by centrifugation for 5 min at 600g at 4°C. The supernatant was removed and remaining cells were re-suspended in the red blood cells (RBC) lysis buffer (MACS), incubated for 10 min and finally centrifuged for 5 min at 600g. After centrifugation, the supernatant was discarded and the pellets were washed with 2% FCS in PBS. The dead cells from the sample were then removed using the MACS dead cell removal kit according to the manufacturer's guidelines (MACS Miltenyi Biotech, Gladbach, Germany). The collected sample was centrifuged and washed twice, each time pelleting the cells before washing in 2% FCS and discarding the supernatant. The cells were re-suspended in 2% FCS in PBS and separated into 100 µl aliquots. The cells were incubated for 30 minutes with the following antibodies SCA-1, PECAM1, PDGFRα (R&D systems, Minneapolis, MN). Prior to FACS analysis the cells were washed twice with 2% FCS in PBS. The cell population was analyzed and sorted by FACS analysis using BD FACS Aria™ Cell Sorter (BD Biosciences), to select the required population based on cell size and surface cell markers. Using compensation samples and isotype controls, the required population was obtained based on desired expression of cell surface markers and were collected in a 15 mL Falcon tube containing cell culture media.

Method S2: Live and dead staining protocol

Calcein AM and Propidium Iodide (PI) was used for live and dead staining. Fibroblast L929 cells (Sigma Aldrich, 04102001-1VL) were injected into the device using hand-held pipettor and were left to attach for 4 hours. The stamp was peeled-off from a 6-well plate and washed once prior to staining. The cells were incubated with 4 µM Calcein AM and 1 uM PI for 1

hour followed by medium replacement. Prior to imaging, the staining efficacy was checked using Olympus IX73 Olympus microscope.

Method S3: Segmentation of EC and SC

Phase contrast movies showing the proliferation and migration of SC and EC where segmented using the Matlab^{TM} Image Processing Toolbox. Phase contrast images were preprocessed with a low pass spatial filter (average of 30×30 pixels) to make image intensity uniform, and then thresholded to segment cells, which have low intensity compared to background. After binary image closure, EC were distinguished from SC by the size and shape of their nuclei using the 'regionprops' function (area and eccentricity); EC have a small dark central region whereas SC with fibroblast morphology where identified by a larger their elongated dark region. Three movies where acquired for each experimental group. Statistical comparisons of EC and SC in homogeneous culture (EC|EC or SC|SC) or co-culture (EC|SC) where compared using a linear regression models which estimated the slope and intercept for log2 transform cell numbers (doublings) versus time. The effect of covariates (co-culture or cell type) on the slope and intercept of growth curves were estimated.

Table S1.Device operating pressure range under various substrates (n=5)

Substrate type	P_{low} *(kPa)*	P_{up} *(kPa)*
PS cell culture dish	*1.032± 0.105*	*7.763± 0.437*
Glass	*0.743± 0.365*	*9.731 ±0.815*
